# Pre-Weaned Calf Rearing on Northern Irish Dairy Farms: Part 1. A Description of Calf Management and Housing Design

**DOI:** 10.3390/ani11071954

**Published:** 2021-06-30

**Authors:** Aaron J Brown, Gillian Scoley, Niamh O’Connell, Jamie Robertson, Amanda Browne, Steven Morrison

**Affiliations:** 1 Agri-Food and Biosciences Institute, Hillsborough BT26 6DR, UK; Gillian.scoley@afbini.gov.uk (G.S.); steven.morrison@afbini.gov.uk (S.M.); 2 Institute for Global Food Security, School of Biological Sciences, Queen’s University, Belfast BT7 1NN, UK; niamh.oconnell@qub.ac.uk; 3 Livestock Management Systems Ltd., Aberdeen AB11 5DE, UK; j.robertson@lms2004.co.uk; 4 Agri-Food and Biosciences Institute, Newforge Lane, Belfast BT9 5PX, UK; Amanda.browne@afbini.gov.uk

**Keywords:** dairy calf, health, performance, survey, calf housing, hygiene

## Abstract

**Simple Summary:**

Calf health, welfare and performance in the first few months of life are important for the lifetime productivity of dairy cows. Therefore, all aspects of management, including nutrition, hygiene, housing and health, should be optimal to maintain productive calves. As there is little information surrounding these areas on Northern Irish dairy farms, this study investigated 66 farms with the objective of describing common trends and identifying factors which have the potential to compromise calf health, welfare and performance. A large degree of variation was seen in calf housing and other management practices across the farms. The majority of housing, in terms of building design and functionality, was identified as sub-optimal and is likely a contributing factor for selection of particular management practices. Regular monitoring of management practices such as measuring quantity of feedstuffs provided and animal live weight allows farmers to assess the effectiveness of their rearing system. However, this study highlighted a lack of appropriate monitoring and measuring in several key areas that present a risk for calf health and performance.

**Abstract:**

The first few months of life are of great importance to the longevity and lifetime performance of dairy cows. The nutrition, environment and healthcare management of heifer calves must be sufficient to minimise exposure to stress and disease and enable them to perform to their genetic potential. Lack of reporting of farm management practices in Northern Ireland (NI) makes it difficult to understand where issues impacting health, welfare and performance may occur in the rearing process. The objective of this study was to investigate housing design and management practices of calves on 66 dairy farms across NI over a 3-month period and also identify areas that may cause high risk of poor health and performance in dairy calves. An initial survey was used to detail housing and management practices, with two subsequent visits to each farm used to collect animal and housing-based measurements linked to hygiene management, animal health and performance. Large variations in key elements such as weaning criteria and method, calf grouping method used, nutritional feed plane, and routine hygiene management were identified. The specification of housing, in particular ventilation and stocking density, was highlighted as a potential limiting factor for calf health and performance. Lack of measurement of nutritional inputs, hygiene management practices and calf performance was observed. This poses a risk to farmers’ ability to ensure the effectiveness of key management strategies and recognise poor calf performance and health.

## 1. Introduction

Management in early life is a fundamental factor enabling dairy calves to reach key performance targets that impact on lifetime productivity [1]. For example, previous research has highlighted the influence of average daily gain (ADG) in the pre-weaned period on the onset of puberty, age at first calving and performance in subsequent lactations [2]. Ensuring that calves attain performance targets requires a multifaceted approach. This not only includes providing adequate nutrition, but also proactive management of their environment and healthcare to minimise the risk of reduced feed efficiency, survivability and compromised welfare.

Physical aspects of the rearing environment, such as ambient and surface temperature, relative humidity, air speed and surface moisture, have been shown to influence calves’ physiological state [3]. For example, combining cold temperature with damp surfaces and raised air speed will exacerbate cold stress in young calves leading to a diversion of energy toward maintenance of thermoregulation. Grouping strategies are also important, and evidence suggests that grouping calves at a young age can improve solid feed intake and calf welfare [4,5]. This strategy also has the potential to greatly reduce labour; however, the increased risk of disease transmission between calves may concern producers [6,7]. Hygiene management is also an important aspect of rearing calves as their immune systems are naïve and infectious pathogen levels can rapidly increase in the calf environment [8]. High levels of bacteria in the pen bedding, air, feeding equipment and the feed are associated with increased calf enteric and respiratory disease and should therefore be avoided [9]. Housing and management during rearing should therefore aim to minimise calf exposure to stress and pathogens, particularly those associated with enteric and respiratory diseases, within the first months of life.

There is evidence of shortfalls in commercial dairy calf rearing practices throughout the UK with previous studies highlighting that health and performance of pre-weaned calves on dairy farms may be compromised. Brickell et al. [10] reported that 15% percent of dairy heifers, born alive, did not reach their first lactation, with a mortality rate of 6.8% within the first 6 months of life. As the dairy heifer accumulates cost throughout the rearing period until first calving, this represents a significant economic loss to farmers. More recently, Hyde et al. [11] found mortality in dairy calves in the UK to be 6% in the first 3 months of life and that changes in mortality were minimal when compared with the 1990s [12]. Improvements to calf health and performance with developments in nutrition and healthcare may be limited by a poor environment, and similarly poor nutrition and healthcare may negate the effects of a good environment. Therefore, all these aspects should be evaluated when assessing calf rearing systems.

Although environmental factors such as ambient temperature have been shown to play a fundamental role in calf mortality [11], many studies only provide limited environmental descriptors when reviewing calf rearing systems. Additionally, systems vary across different regions. Numerous surveys have been conducted in US regions where temperatures regularly fall below 0 °C and barn design is typically consistent across farms [13,14,15,16]. Klein-Jöbstl et al. [17] noted similar consistency in areas such as colostrum management, housing and feeding procedures within Austrian dairy farms. In the UK however, there is a dearth of information linking calf performance and health with routine calf rearing practice and housing design. Moreover, there is no information surrounding calf rearing systems in Northern Ireland (NI).

Information on current calf management practices is required to determine the major pitfalls in the rearing phase on commercial farms, providing direction for future research. Understanding the motivation behind particular farming practices will provide insight to solutions required to improve uptake of updated recommendations by producers. In order to address the knowledge gap, a survey of calf rearing was completed on dairy farms within NI. The key objectives were to describe common approaches to housing and management and identify potential areas of weakness that may lead to poor calf health and performance. In particular, the specifications and condition of housing used, and management of nutrition and disease were evaluated.

## 2. Materials and Methods

### 2.1. Farm Selection and Enrolment

Seventy-five farms were chosen for potential participation in the survey, this equating to approximately 3% of total dairy farm businesses in NI [18]. In order to be eligible for inclusion, farms had to be recording for NI national benchmark figures and have a herd size of more than 60 dairy cows. This was to ensure that adequate farm financial and physical data were available and that the rearing operation was large enough to require comparable calf rearing facilities, respectively. Within each of the six counties in NI, farms were stratified by size and a random sample was taken from each stratum to ensure appropriate herd size and geographical distribution. Farmers were first contacted by letter to indicate that farm recruitment would be occurring and following this they were contacted by phone to confirm their willingness to participate and to schedule the first farm visit. Of the 75 farms selected, 66 agreed to participate. 

### 2.2. Farm Visits

Each participant was visited 3 times, over a 3-week period; the entire sampling period was carried out between the 18 January 2019 and 2 May 2019. Data collection took place through a combination of a questionnaire with the farm manager or main calf rearer, a review of farm records and on-farm housing and hygiene measurements collected by trained assessors. Prior to the beginning of data collection, assessors were trained in each element of data collection by the authors on a non-participating farm (CAFRE Greenmount dairy farm, Northern Ireland) prior to the study, to ensure consistency in of data collection techniques. The face-to-face questionnaire (available as Supplementary Material) was completed on the first farm visit and covered nutrition, healthcare, and housing practice from birth until weaning, incidences of calf morbidity and mortality over the current calving season, and farm demographic information. Number of calf births, calf mortality between 0 and 2 days of age, and from 2 days of age to weaning, as well as the number of calves treated for scour and pneumonia were ascertained from farm records and grouped by month. Other disease information was recorded in the survey questionnaire. Housing and hygiene measurements were carried out on all of the visits. Each second visit was arranged for the earliest convenient date after the first (average of 8 days after), and the third visit took place two weeks after the second visit.

### 2.3. Nutritional Measurements

The nutritional management of pre-weaned calves until the age of 13 weeks was recorded. The level, concentration and nutritional composition of liquid and solid feed offered to calves were recorded to calculate dietary metabolisable energy content (ME, MJ/kg DM) [19]. Protein and fat content of cow’s milk fed to calves were ascertained using milk recording data collected within the month of the first farm visit. ME (MJ/kg DM) of cow’s milk was calculated using methods described in the [19] where lactose was taken as 4.85% [19]. Farmers were asked to demonstrate routine milk replacer mixing by preparing a perceived allocation for a calf or given number of calves. This was then weighed by technicians to determine the accuracy of the actual quantity of milk powder against the target amount. Age of access to water and forage as well as method of presentation for the pre-weaned period were also recorded.

### 2.4. Hygiene Measurements

Hygiene in the calving pens was categorised as good, intermediate or poor using the following classifications. Hygiene was considered good when well strawed, no damp areas and with no sick cows. Poor hygiene was recorded when there was a clear build-up of dirt at animal height, engrained dirt in walls and floors, and presence of use for sick cows. Pens were classed as moderate where there was a low level of dirt build up, although mostly well strawed and no sick cows. Calving pens with porous surfaces at calf height (e.g., non-rendered stone walls), significantly cracked concrete floors, and poor access for machinery were described as not easy to clean. Pens were described as easy to clean where no porous surfaces were present, floors were intact and there was easy access for machinery.

### 2.5. Housing Measurements

#### 2.5.1. House and Pen Dimensions

The area and volume of calf buildings and all calf pens, and the eaves and ridge height of calf buildings were recorded (Draper Laser Distance Measurer; LDM-40M, Draper Tools Ltd., Eastleigh, UK). The materials of calf house roofs, walls, and pen walls and floors were recorded. The slope of representative locations in pen floors were measured in up to three pens using a digital spirit level (Gain Express G0182105-JY4, Gain Express Holdings Ltd., Hong Kong) orientated to find the maximum slope. 

#### 2.5.2. Ventilation

Ventilation was classified as natural or mechanical. The length and width of outlets above eaves height were measured directly or estimated from photographs, and the area of inlet in each sidewall was calculated from measured surfaces and known porosity of standard cladding materials [20]. The ventilation requirement (K) of each building was calculated for the maximum number of calves placed in the building at one time as stated by the farmer, using the methodology described by Bruce [21]. The minimum outlet and inlet areas derived from K were compared with the actual inlet and outlet areas (KO) to provide an assessment of natural ventilation capacity. Manufactured calf hutches (*n* = 5) were excluded from natural ventilation calculations, meaning calculations were completed for 61 calf houses. The ratio K:KO was used to describe the competence of inlet and outlet areas to provide natural ventilation for the housed animals. Ratios of ≥1:1 were classed as good, 1:1 to 1:0.75 was classed as average and <1:0.75 was classed as poor. The distribution of inlets around each building was described as good, average or poor based on the ratio of the derived minimum inlet area between the two main sidewalls (good = ratio of 40:60 or closer, average = 30:70 to 40:60, poor = 30:70 or wider). 

#### 2.5.3. Lighting

Spot measurements of natural and artificial light were taken at calf head height in 3 pens on each farm (Extech 45170 Multimeter, FLIR Commercial Systems Inc., Nashua, NH, USA) between 10:00 h and 16:00 h. The area of natural lighting in the roof was recorded as a percentage of the total roof area of the calf building. This was determined by dividing the number of transparent roof sheets by the total number of roof sheets.

### 2.6. Statistical Analysis

Data were entered into Genstat^®^ (version 21, VSN International Ltd., Hemel Hempstead, UK). For each continuous variable summary statistics were calculated to include the number of observations recorded, the mean and the standard deviation. For the categorical variables a table of counts showing the number of observations for each level of the variable in question and also a table showing the percentage of each level over the total in each case was produced.

## 3. Results

### 3.1. Farm Demographics

The median herd size of farms was 124 cows (ranging from 66 to 400). Holstein was the predominant breed of cow (78.8%) within the herds (Table 1). Average 305-day yield production of the herds ranged from 5000 L to 11,000 L with a mean of 8000 L. Calving patterns differed greatly across farms, with the majority calving from approximately September to March (47%) or calving all year round (40.9%). The spread of the calving period ranged from 77 to 365 days with an average of 272.2 days. Within this dataset, the range of herd calving patterns and herd sizes meant that the requirements in relation to each farm’s calf accommodation and management would also vary accordingly.

The average age of the main person responsible for rearing of calves (e.g., farm manager, owner or main calf rearer) was 49 years, ranging from 23 to 82 years, and 86.4% were male. The average amount of time that this person spent per day feeding calves and completing other calf related tasks was 125.4 min. A second person that assisted in calf rearing was available on 42.4% of the farms.

### 3.2. Nutritional Management

Within the farms surveyed, only 3% of farms left calves to suckle the dam for their first feed. This suggests that many farms were taking a direct approach to ensuring calves receive adequate colostrum by manually feeding it to ensure successful passive transfer of immunity [22]. Although the majority of farms (77.2%) fed 3 litres or more of colostrum in the first feed, only a small number (13.6%) regularly tested it to determine the quality, meaning that there is still a risk of calves having failure of passive transfer (FPT) if quality is low [23].

Of the 66 farms surveyed, 81.8% fed calves milk replacer throughout the pre-weaned period whereas 18.2% only fed cow’s milk. The main method of feeding calves was through single teat feeders, likely due to the fact that 62.1% of the farms were not grouping calves until at least 1 week of age. Automatic feeders (AMF) were used on 21.2% of the farms which is similar to other regions [24]. The majority (57.1%) of farms using AMF calibrated them annually, whereas 7.1% did not routinely calibrate them. A variety of milk replacer products (*n* = 25) were used on farms, with protein and fat levels ranging from 20% to 26%, and 16% to 22%, respectively (Table 2). Protein content of cow’s milk fed ranged from 3.14% to 3.60%, with butterfat content ranging from 3.71% to 4.59%. The mean metabolisable energy (ME) (MJ/kg DM) of milk replacers fed was 19.16 MJ/kg DM whereas the mean ME of cow’s milk fed was 24.85 MJ/kg DM. Milk replacer concentration was an average of 152 g/L, and the majority of samples (84.6% of 26 farms) weighed by technicians were within 90% to 110% of the targeted amount of milk replacer powder. Across the farms in this study, median peak milk volume allowance was 6 L/day (ranging from 4 L/day to 8 L/day), where there was a median of 7 days to peak milk volume. 

Calf starter feed was offered to calves from as early as birth and the mean first age of being offered this feed was 5 days. The majority of farms (78.8%) did not measure starter feed offered to pre-weaned calves. On the remaining farms, the mean quantity offered per day at the point of weaning was 2.1 kg, ranging from 1.5 to 3.5 kg. Protein content ranged from 16% to 21% and the average ME (MJ/kg) of starter was 9.83 MJ/kg.

Water was offered at birth on 68.2% of farms, and the mean first age of offering was 4 days. The method of offering water varied across the pre-weaning period between and within farms, with bucket being the predominant (80%) method in the first week of life and free flow drinkers by 5 weeks of life (57.6%). Forage was first offered to calves at an average of 4 days of age. Straw (excluding bedding) was the main forage type (65.4% of farms) offered to pre-weaned calves. Alternative forages offered were hay (25%), haylage (1.9%), lucerne (1.9%) and straw within a mixed ration (1.9%). Forage was fed through racks or troughs, which varied within farms depending on the age of the animal; however, 20% of farms perceived bedding to be a suitable source of forage for calves and did not offer additional forage.

### 3.3. Physical Management

The average age at the start of weaning was 57.7 days and a variety of weaning criteria were used (Table 3), the most common being age (33.3%). Within this dataset, 13.6% of farms weaned calves by concentrate intake alone, and farms that used this as a factor tended to quote intakes of 2 kg per day as the minimum threshold for weaning. Farms also weaned calves depending on visual appearance alone (7.6%) or on a combination of two or more factors (36.4%). The most common weaning method was abrupt weaning (31.8%), this followed by a 3-step reduction in milk volume (28.8) and then a 2-step reduction in milk (27.3%) volume.

As a means of improving calf thermoregulation, calf jackets were used on farms routinely (28.8%), during situations of cold weather (19.7%) or where calves were small or sick (21.2). Of the farms that used calf jackets, 34.7% did not wash them between calves, presenting a risk of pathogen transmission to younger calves. Heat lamps were used on a lower proportion of farms (50%), with only 4.6% using them routinely.

### 3.4. Hygiene Management

Single calving pens were in use on 73% of farms. Bedding was added daily or after every calving on 61% of farms, with 21% of farms adding bedding 3 times per week or less. Only 15% of calving pens were cleaned out after every calving or every other calving, a further 33% weekly or fortnightly, and more than half (52%) had a cleaning interval of one month or more. Hygiene in the calving pens at the time of survey visit were scored as 32% good, 47% moderate and 21% poor, with 24% of pens considered not easy to clean. Furthermore, 25.8% of the farms reported using calving pens for sick cows. 

The median number of days between cleanings in single and group calf pens was 28 and 31.5, respectively. Cleaning frequency of feeding equipment (buckets and teat feeders) ranged from twice per day to every 84 days with a median of every 2 days. Feed buckets were cleaned when the respective calf was weaned on 18.2% of the farms. Automatic feeders were cleaned on average every 7.7 days, although 50% of farms using AMF did not routinely clean teats. The range of hygiene routines reflects the diversity of the farms sampled in this study. The majority of farms (83.3%) used disinfectant when cleaning however, 26.4% of these farms did not measure the concentration of disinfectant. Approximately 41% of the farms did not use or measure the concentration of disinfectant, this in turn suggests that there was a high risk of their pen hygiene management not being effective in completely removing pathogens. 

The majority of farms did not have designated areas to wash (65.2%) and dry (77.3%) feeding equipment (Table 4), making it difficult for them to consistently maintain effective hygiene practice throughout the calving period. Approximately 29% of farms had calves in the calf house throughout the year, so thoroughly cleaning housing would have been difficult. 

### 3.5. Housing Design and Management

A large diversity in calf housing was seen in farms across the dataset. Hutches were used as primary calf housing on 7.5% of farms. The majority of calf houses (92.4%) were classed as naturally ventilated as they did not have mechanical ventilation present; however, a large proportion had inadequate inlet and outlet areas (53%) to achieve natural ventilation (Table 5). Additionally, 53% of the main calf houses were adjoined to other livestock buildings meaning that natural ventilation would be further compromised. Main building volume per calf ranged from 6.2 m^3^ to 75.3 m^3^, with an average of 20.6 m^3^, lower than calf house volumes (mean of 36.4 m^3^) surveyed in the US [14].

The most common time to group calves was between 1 and 4 weeks of age (39.4%; median age at grouping was 14 days) although there was a large variety across the dataset, ranging from birth to 64 days of age. Approximately one-quarter of farms surveyed (28.8%) did not give a response to reason for time of grouping; however, the most common responses given related to ease of management (22.7%), age of the calves (19.7%) and availability of space (15.2%). 

Space allowance (S) within calf pens ranged from 0.9 m^2^/calf to 4.9 m^2^/calf with an average of 2.3 m^2^/calf, more specifically average SA in single pens and group pens was 1.5 m^2^/calf and 3.4 m^2^/calf, respectively. Light intensity at calf level within calf housing ranged from 8.0 lux to 5156.7 lux (where lights were turned on) with a median of 256.7 lux. Average light intensity was below 100 lux on 19.7% of farms. 

Straw was the predominant substrate used to bed calves on farms (92.4%), likely due to its established ability to insulate and provide nesting for young calves [25]. In this study, an attempt was made to measure the slope of calf pen floors however the presence of deep bedding and calves in the pen made this challenging. Although 57.6% of farms had solid concrete floors under calves, the ability of these to sufficiently drain urine, spilled milk and water is determined by the slope and could vary significantly. Of the 99 successful measurements taken on 52 farms, the median slope recorded was 1.1°, ranging from 0° to 5.2°. 

### 3.6. Calf Health and Disease Management

Separation of sick calves is a well-established practice to minimise the risk of disease spread to susceptible calves [26]. Although 42.4% of the farms isolated calves at first signs of sickness, 24.4% of farms did not separate calves until severe signs of sickness and 18.2% did not isolate calves at all, potentially enabling transmission of pathogens between calves.

According to farmers’ records, infectious bovine rhinotracheitis (IBR) was the most common (21.2%) on farms within the last three years (Table 6). Bovine Viral Diarrhoea (BVD) and Leptospirosis were the diseases most commonly vaccinated against on farms (75.6% and 72.7%, respectively) (Table 7). In terms of biosecurity, 42.4% of the farms had visitor foot dips present at the entrance. However, 53.5% of these did not routinely replenish them, suggesting that these foot dips would not likely be effective as a biosecurity measure [27]. Of the other farms that had foot dips present, 28.5% replenished them routinely within two weeks whereas 14.4% routinely changed them less frequently than every two weeks. A much smaller percentage of farms (9.1%) used foot dips as a measure of biosecurity within farm and only 33% of these regularly replaced them within two weeks. As an alternative biosecurity measure, 21.2% of farms operated a closed herd policy where no cattle were purchased into the farm.

## 4. Discussion

### 4.1. Farm Demographics

The herd sizes observed in this study were typically smaller than those reported in the US and other parts of the UK [11,15] but larger than those surveyed in Europe [17,28]. Several studies have associated larger herd sizes with increased calf mortality [28,29]; however, Murray et al. [30] found conflicting evidence. On average, farms were calving for over three quarters of the year. Only a small percentage of herds (6.1%) were spring calving with the aim of a short calving period, in contrast with that typically seen in the Republic of Ireland [31,32]. Increasing the spread of the calving season has been associated with an increase in calf mortality [30] and calves born in winter or spring months are at greater risk of morbidity and mortality [33,34].

### 4.2. Nutritional Management

The feeding of cow’s milk to calves, as reported on 18.2% of farms surveyed, allows calves to benefit from the increased fat content and ME content compared to milk replacer [19], but there is a risk of disease transmission and inconsistency in milk components [35]. Much research has been completed in the area of milk volume fed to calves and increased peak volume of milk is associated with improved calf performance, health and welfare [36,37]. Peak milk volume ranged from 4 L to 8 L per day across farms and the difference in energy intake may have significantly impacted performance of calves. However, the median time to peak milk allowance of 7 days was comparatively less than in calf diets reported by Jorgensen et al. [37], where the mean was 18 ± 11.4 days and increasing the number of days to peak allowance was associated with poorer calf health. 

One in five farms did not offer calves forage. Forage provision in the pre-weaned period increases starter intake in the same time period, with a subsequent increase in calf performance [38]. Furthermore, the provision of alternative forages may reduce the intake of straw bedding, which presents one of the largest sources of pathogens in the environment of calves and is therefore a risk factor for disease [26,39]. The simple addition of forage to pre-weaned calves would not only reduce the risk of calves consuming contaminated bedding, but provide an opportunity for novel behaviours that reduce cross-suckling [40] and increase the consumption of starter intake which is important for effective and non-disruptive weaning [41]. The adequate consumption of starter at the beginning of weaning has been shown to be more important than the weaning method employed [42].

Age is the simplest unit of measurement for weaning and was used as the sole reason for weaning on a third of farms. However, controlled research suggest that calves should be eating approximately 1.5–2 kg of starter feed before being fully weaned [43]. Weaning before calves are consuming this quantity of concentrate has been indicated to result in an increase in stress and poor performance as they are not fully adapted to a post-weaned diet [42,44,45]. Therefore, weaning by age alone may present a risk to calf performance and health in weaned calves. Industry has pointed to a target of doubling birthweight by the point of weaning [46], which has put some emphasis on live weight as criteria for weaning. However, only 9.1% of farms in this study weaned by weight alone. This may reflect a lack of accurate methods for accurate weighing of calves on farms which is supported by the fact that very few of the farms had an appropriate equipment for weighing calves (weigh scales or chest girth band) [47]. Of farms surveyed, 31.8% chose to wean calves abruptly with no prior reduction in milk volume. Abrupt weaning has previously been linked with increased signs of hunger and cross suckling due to sudden reduction in energy intake [48,49]. Where there has been failure to establish calves on sufficient concentrate feed prior to weaning, stalls or reduction in growth performance have also been observed [50,51]. The variety of weaning criteria and methods observed across farms suggest lack of clear guidance and understanding surrounding the requirements of the weaned calf, or the necessary tools required to measure calves’ suitability for weaning.

### 4.3. Hygiene and Housing Management

Poor calving pen hygiene has been established as a factor in the transmission of cryptosporidium to calves [52] as well as playing a small role in early lactation uterine infections in the dam such as endometritis [53]. Increased exposure to manure of adult cattle as well as exposure to adult cows other than the dam has been shown to increase the risk of Johne’s disease transmission to newborn calves [54]. The use of multiple cow calving pens may also increase the risk of Johne’s disease transmission, although other factors such as the pooling and contamination of colostrum are also known to play a role [55]. Within the present study, 25% of farms surveyed housed sick cows in calving pens. As indicated above, this suggests there is significant potential for accumulation of pathogens within calving pens and therefore an increased risk of disease transmission to newborn calves. Increasing awareness to the potential disease risks originating from the calving pen may increase uptake of more bio-secure and hygienic practices. However, considering the role of available space and time in other management decisions within this study, such as grouping and weaning, the design of facilities may be a limiting factor in the hygiene management of calving pens.

One of the major challenges for calf houses is the duration of use throughout the year, for example 89.4% of farms calved cows over 9 months of the year or more. The sustained arrival of new calves over an extended period such as this makes it difficult for farmers to find opportunity to clean. Additionally, the continuous flow of calves through calf housing without breaks presents the challenge of young calves being exposed to older calves [56]. The use of modular housing (where the management of one pen can be independent of another) has the potential to allow farms to maintain hygiene practices where there is constant demand for calf accommodation. Four out of five of the farms using hutches availed of this benefit, with cleaning occurring after each use. The proportion of farms using hutches (7.6%) was much lower than in countries such as the US where hutches are commonplace [57]. On many farms, the lack of routine cleaning regimes may be a factor in poor calf health and performance.

Across the dataset, ventilation of calf houses was poor; a small number of farms used mechanical ventilation (7.6%) and a large proportion had limited natural ventilation design present in calf housing (53%). Adjoining calf houses to other buildings not only risks impacting ventilation [14], but in many cases would also risk airborne pathogens being transferred from older animals to calves that would be detrimental to calf health [56]. Pre-weaned calves were forced to share airspace with older, post-weaned animals in 51.5% of the calf houses. Calf houses were adjoined to other buildings on 53% of farms. Even where design of housing allows natural ventilation, it is suggested calves do not produce sufficient heat to drive thermal buoyancy meaning that during times of low wind speed, ventilation will be inadequate [56]. Very little recent research has been carried out in the natural ventilation of cattle housing within the UK climate. Additionally, anecdotal evidence would suggest that mechanical ventilation is not commonly used in calf housing in the UK. More research should be conducted to assess the ability of naturally ventilated housing to meet the ventilation requirements of pre-weaned calves as this will vary with geographical area, topography, building orientation, situation and cladding design [58,59]. 

Although the average calf house volume (20.6 m^3^) was lower than those surveyed in the US by Lago et al. [14], there was a similar range whereby the volume of many buildings was excessively higher than the minimal recommendations of 6 m^3^/calf [60]. The range of 69.1 m^3^ between the smallest and largest volumes per calf may have an impact on ventilation rates [14].

The average SA in calf pens was higher for group pens (3.4 m^2^/calf) than single pens (1.5 m^2^/calf). Research assessing calf barn design on airborne bacterial density has concluded that SA has a large impact on calf house air quality and has recommended a SA of 3.0 m^2^ per calf [14], which has also been suggested to improve calf welfare [61]. This highlights that group pens within this study that are typically constructed within the housing are of more adequate space than the single pens being used. As many of the single pens used on farms surveyed were commercially available products, achieving an overall increase in SA would be heavily dependent on the adoption of larger pen designs by manufacturers. 

Although little research has been conducted on the impact of light intensity on calf performance and welfare, levels below 100 lux have been associated with reduced feeding and social behaviour and increased lying behaviour [62]. As the median light level was 256.7 lux, it could be considered that light was not a limiting factor on farms surveyed. The provision of natural light is often thought to be productive in the elimination of pathogens although this has been seen to vary across micro-organisms and the intensity of sunlight [63].

Ease of management was the primary reason for the age of grouping calves on 22.7% of farms surveyed. Labour associated with calf rearing is a challenge for many producers and grouping calves earlier is widely recognised to ease management, regardless of herd size [64]. The time gained by easier management may be of benefit to farmers and allow other tasks such as cleaning to be carried out more thoroughly. The finding that 15.2% of farms grouped calves based on the availability of space suggests that the suitability of housing may have been a limiting factor in calf rearing. Further research could investigate relationships between housing system and grouping management. 

Jackets and heat lamps are typically used on farms to help calves maintain an appropriate body temperature [65]. A majority of farms (74.2%) used calf jackets in some capacity, despite the fact that research has shown no clear benefit to improving performance or health [65,66]. As there may be variation in what temperatures are classed as cold by individual farmers, the use of thermometers in the calf house would provide a more accurate method of monitoring temperature and placing a minimum threshold under which calves are provided with jackets. Heat lamps were predominately used for sick or small calves but the majority (54.4%) of farms did not use them at all. Young calves prefer heat supplemented areas during cold periods so the provision of heat lamps to pre-weaned calves could be a benefit to their welfare and health [67]. Straw plays a large role in the rearing of dairy calves in Northern Ireland, between its uses mainly as bedding, but also as a source of forage for early rumen development. As arable and horticultural crops make up just 4.3% of the farmed land area [18] as opposed to 26.9% across the UK [68], livestock farms in Northern Ireland are heavily reliant upon imported straw, the cost of which is volatile. This presents the risk of high cost of bedding and anecdotal evidence would suggest that this may cause reluctance to substantially bed calves to maintain a high level of hygiene and provide suitable nesting and thermal protection. The ability of substrate to maintain a dry bed is influenced by the specification of the floor beneath it [56]. The use of perforated (slats) or permeable floor materials (stones or woodchip) on farms may increase drainage efficiency, but there may be implications on ease of thorough cleaning of the surface between calves, although no research has evaluated previously this. Considering the potential influence drainage may have in the calf environment [69], further research into this area is required. 

### 4.4. Health Management

In this study, the majority of farmers vaccinating for BVD and Leptospirosis was similar to that of a previous survey of UK cattle health management, as was the percentage of farms operating a closed herd policy [70]. Although the number of farms that reported vaccinating for Rotavirus (43.9%), Coronavirus (34.8%) and E. coli K99 (22.7%) differed, there is evidence that the only commercially available calf enteritis vaccines cover all the aforementioned diseases. This discrepancy may suggest that some farmers are not fully aware of the targeted diseases of the vaccines they purchase. This may highlight deficiencies in knowledge transfer from the farm’s veterinary service. For future studies, it may be beneficial to discuss questions pertaining to the health and vaccination management with the farm veterinarian.

Results of the present study have highlighted areas of increased risk within numerous aspects of calf rearing management. The adage ‘measure to manage’ is often used in agriculture but in this survey, lack of measurement has been demonstrated within the nutrition, hygiene and physical management of calves. The majority of farms did not measure colostrum quality and starter intake in calves. Adequate intake of high-quality colostrum is paramount to the health of neonatal calves and measuring colostrum quality is an inexpensive and relatively quick method of ensuring calves are ensuring more than 10 mg/mL of immunoglobulins [23]. Similarly, sufficient intake of solid starter feed is necessary to maintain energy intake and minimize stress in the post-weaning period so that calves continue to thrive and are not at greater risk of diseases like pneumonia [42,71]. Additionally, only a small percentage of farms weighed calves, which is a vital monitoring requirement to ensure they are reaching key performance targets. Failure to meet performance targets throughout the rear period may lead to a greater age at conception and first calving. As the cost of rearing is approximately GBP 1819/heifer [72], it is expensive to wait until calving before identifying issues stemming from the rearing phase [73]. The ability to categorise performance in the pre- and post-weaned periods allows producers to evaluate the effectiveness of their nutrition, health and rearing environment in enabling optimum growth in calves. Regarding hygiene, a large proportion of the farms did not use or measure disinfectant when cleaning calf pens, meaning there is a likelihood that their hygiene management was not effective. If the level of cleaning between groups of calves is insufficient there is a risk of pathogens being transmitted from calves to others subsequently housed in the same pens [74]. Furthermore, there as a large proportion of farms were rearing calves for 9 or more months of the year, there would likely be a gradual increase in the level of microbes in the calf house potentially increasing the risk of calf enteritis [17].

## 5. Conclusions

A large degree of variation was observed in nutrition, hygiene and housing management across farms in this study. Observations within the study that may negatively impact calf performance and health included small space allowance for calves, suboptimal ventilation, and inadequate drainage and it is suggested that the housing management of pre-weaned calf housing in Northern Ireland may be a limiting factor for calf performance and health. Additionally, the specification and condition of physical housing on farms may limit the ability of farmers to adopt improved housing and hygiene practice. Increased availability of products and materials that facilitate simpler management of calves and ease of cleaning may improve uptake of best practice.

Sub-optimal nutritional and rearing environment can be identified through measuring of calf growth performance; however, this was not carried out routinely on the majority of farms. Increasing the monitoring of calf liveweight would provide a cost effective indication of performance efficiency in pre-weaned period, which allows changes to be made long before the animal reaches first calving. Similarly, regular measuring of inputs would increase the accuracy of nutritional and hygiene factors that impact rearing success. By measuring these inputs and outputs, farmers would be in a better position to ensure their system is enabling the rearing of healthy, productive calves.

This paper identifies several weaknesses as well as strengths in dairy calf management in NI. Describing these presents the opportunity for research and knowledge transfer to target key areas within calf rearing to the benefit of calf health, welfare and performance, and subsequently dairy farm resilience and profitability.

## Figures and Tables

**Table 1:** Dairy herd demographic information of the 66 participating farms.

Variable	Response	%
**Main cow breed**	Holstein	78.8
	Friesian	6.1
	Holstein crossbred	7.6
	Fleckvieh	4.6
	Jersey	1.5
	Montbeliarde	1.5
**Calving Season**	All year round	40.9
	Autumn–Spring ^1^	47.0
	Autumn block ^2^	4.6
	Spring block ^3^	6.1
	Spring–Winter ^4^	1.5

^1^ Calving taking place from September/October until March/April; ^2^ Calving taking place from August/September until December; ^3^ Calving taking place from January/February until March; ^4^ Calving taking place from April until December.

**Table 2:** Liquid feed nutrition of pre-weaned calves.

Variable	Mean	Maximum	Minimum	SD
Crude protein content (%)	22.5	26.0	20.0	1.6
Crude fat content (%)	18.1	22.0	16.0	1.3
MR ^1^ ME content (MJ/kg DM)	19.2	20.2	18.0	0.6
Age calves first offered MR ^1^	6.7	28.0	1.0	4.6
MR ^1^ Concentration (g/L)	149.5	225.0	24.3	34.5
Peak milk volume (L/day)	5.5	8.0	4.0	1.1
Time to peak milk volume (days)	11.2	49.0	0.0	12.4

^1^ Milk replacer.

**Table 3:** Physical management of pre-weaned calves.

Variable	Response	%
Calf’s navel dipped at birth	Yes	71.2
	No	21.2
	Sometimes	7.6
Calf dried at birth	Yes	7.6
	No	87.9
	Sometimes	4.6
Colostrum quality tested	Yes	13.6
	No	74.2
	Sometimes	12.1
Quantity of first colostrum feed	Less than 3 L	19.7
	3 L to 4 L	33.3
	4 L or more	43.9
	Unknown by farmer	3.0
Weaning criteria	Age	33.3
	Concentrate Intake	13.6
	Size	7.6
	Weight	9.1
	Two or more factors	36.4
Weaning Method	Abrupt	31.8
	2 Step ^1^	27.3
	3 Step ^1^	28.8
	4 Step ^1^	3.0
	Gradual ^2^	12.1
Milk feed method used	Automatic feeder	21.2
	Single teat feeder	51.5
	Group teat feeder	28.8
	Bucket	37.8
	Trough	16.7
Use of calf jackets	Cold temperatures ^3^	19.7
	Calves with increased vulnerability ^4^	21.2
	Cold temperatures or increased vulnerability	4.6
Use of heat lamps	Cold temperatures ^3^	6.1
	Calves with increased vulnerability ^4^	34.9
	Cold temperatures or increased vulnerability	4.6

^1^ Liquid feed allowance reduced in 2, 3 or 4 predetermined steps, respectively; **^2^** Liquid feed allowance reduced gradually over a period of days; ^3^ Cold environmental temperature as determined by farmer; ^4^ Small or sick calves as determined by the farmer.

**Table 4:** Hygiene management of pre-weaned calves.

Variable	Response	%
Reason for cleaning frequency	Availability of bedding	3.0
	To maintain hygiene	33.3
	Suitable practicality	37.9
	Routine management	18.2
	Time availability	7.6
	Unknown	3.0
Disinfectant used in pen cleaning	Yes	83.3
	No	16.7
Disinfectant concentration measured	Yes	73.6
	No	26.4
Separated cleaning area for feeding equipment	Yes	34.8
	No	65.2
Separated drying area for feeding equipment	Yes	22.7
	No	77.3
Use of calf house when no calves present	Rested	63.6
	Storage	3.0
	Always rearing calves	28.8
	Other livestock	4.5

**Table 5:** Physical housing parameters.

Variable	Response	%
Type of Housing used	Indoor individual pens only	9.1
	Indoor group pens only	21.2
	Outdoor hutches only	1.5
	Variety of housing	68.2
Solid pen divisions	All pens	15.2
	Single pens only	22.7
	Starter group pens only	3.0
	None of the pens	57.6
Main floor materials	Concrete	57.6
	Concrete and slats	22.7
	Concrete and stones	1.5
	Slats	9.1
	Stones	7.6
	Woodchip	1.5
Bedding type	Straw	92.4
	Straw and sawdust	4.5
	Baled rushes	1.5
	No bedding	1.5
Ventilation	Mechanical	7.6
	Natural	92.4
K:KO ^1^	Good	28.8
	Average	10.6
	Poor	53.0
Ventilation inlet distribution	Good	27.3
	Average	16.7
	Poor	56.0
Reason for grouping calves	Age	19.7
	Age and ease of management	1.5
	Ease of management	22.7
	Put on AMF	10.6
	Availability of space	15.2
	After vaccination	1.5
	Unknown	28.8

^1^ Proportion of actual ventilation outlet area to required ventilation outlet area, 61 calf houses.

**Table 6:** Farm disease status.

Farmer Reported Diseases Present on Farm	% of Farms		
	Yes ^1^	No ^2^	Don’t Know
Infectious Bovine Rhinotracheitis (IBR)	21.2	48.5	30.3
Bovine Viral Diarrhoea (BVD)	19.7	59.1	21.2
Rotavirus	19.7	34.8	45.5
Coronavirus	7.6	40.9	51.5
E. coli K99	12.1	31.8	56.1
Salmonella	16.7	45.5	37.9
Leptospirosis	7.6	50	42.4
Mycoplasma Bovis	6.1	34.8	59.1

^1^ Positive Cases in the last 3 years; ^2^ Tested negative in the past 3 years.

**Table 7:** Farm health and disease management.

Variable	Response	% of Farms
Farms vaccinating for specific diseases	IBR	31.8
	BVD	75.6
	Rotavirus	43.9
	Coronavirus	34.8
	*E. coli* K99	22.7
	Salmonella	33.3
	Leptospirosis on farm	72.7
	Respiratory disease (calves)	34.8
Preventive measures undertaken by farms	Closed herd	21.2
	Visitors prevented access to calf house	51.5
	Foot dip present at farm entrance	42.4
	Foot dip present at calfhouse entrance	9.1

## Data Availability

The data presented in this study is available on request from the corresponding author. The data are not publicly available due to the General Data Protection Regulation 2016/679.

## Supplementary Materials

The following are available online at https://www.mdpi.com/article/10.3390/ani11071954/s1, Document S1: Calf rearing survey.
