# Supplementary material for: Pre-Weaned Calf Rearing on Northern Irish Dairy Farms: Part 1. A Description of Calf Management and Housing Design
Source: Animals (Basel). 2021 Jun 30;11(7):1954. doi: 10.3390/ani11071954 (PMC8300315; doi:10.3390/ani11071954)
Supplement: Supplementary file 1 [file animals-11-01954-s001.zip › animals-1225213-supplementary.pdf]

Brown et al., S1: Calf rearing survey

Background Information

**Farmer code:**										
**Address:**							GPS Code:			
**Date and time survey conducted:**	_____/_______/______ @ _______:_________									
**Name of team member conducting survey:**										
**Weather at time of visit:**	Temp °C			Wind Direction			N / E / S / W			
	R Humidity %			Wind Speed m/s						
	Outside light level lux:									
	Rain	None			Light			Heavy		
See Visuals	Cloud Cover	0	1	2	3	4	5	6	7	8

Dairy herd information

**No of lactating cow/heifer breeds on the farm:**	HOL	JER	FR	MO	FKV	Other ________________
**COMPLETE CALVING PROFILE (n = number of each breed)**	n= _____	n= _____	n= _____	n= _____	n= _____	n= ___________
**Average production rates: (L/305 day lactation)**			**Current TB Status**		Open / Closed	
**Calving Season (date range)**						
**No of heifers calving into herd per year**			*Expanding cow numbers*		Y	N

Calf and birth management

**Calving pen**	Single n= ________	Communal n= _____	Outside Calving	None
**Size** :	__________m X ____________m	__________m X ____________m	**Comment**: *(e.g. max cows in communal pen, single pens merged?)*	
**Bedding frequency**		**Cleaning out frequency**		

**Comment** *(calving pen used for other purposes as well?/comment on usability/useful good practices):*								
**Hygiene in Calving Pen**	**Good** *Well strawed, no damp areas, no sick cows*			**Moderate** *Low level of dirt build up, mostly well strawed, no sick cows*	**Poor** *Clear build-up of dirt at animal level; engrained dirt, used for sick cows*			
**Are pens easy to clean** *No = presence of porous surfaces at calf height, significantly cracked concrete, poor access*	Y		N	**Clean calving pen between calvings**	Y		N	
**Navel disinfected within 2hrs?**	Y	N	Sometimes	**Calf actively dried**	Y	N	Sometimes	
**Colostrum quality tested?**	Y	N	Sometimes	**Quantity fed in first feed**	______________ litres			
**How quickly are the calves fed after birth?**	*(Is calf left to suck, how long is it left with dam?)*							

<u>Calf House (Tick all relevant)</u> CREATE DIAGRAM ON GRIDLINED PAPER

**Type of calf house (tick all relevant)**	Inside Individual pen	Inside Group Pen	Inside Individual Hutch	Outside Individual Hutch	Outside Group Hutch/Igloo	Outside Group Hutch/ Igloo with sheltered access
	n= _______	n= ______	n= _______	n= _______	n= _______	n= _______
**Size of main calf house (L X W)**	________________m X ________________m			☐	DIAGRAM & PHOTOS	
**Size of other calf houses**	__________m X ___________m	__________m X __________m	__________m X __________m	__________m X __________m	__________m X __________m	
**Comment:**						
**Type of pens**	Individual	Group	Other ________________________			
	n = _______	n= _______	n= ____________			
**No. of calves on milk kept up to weaning per year?**	Male =	Female =	**No. of calves on milk at visit?**	Male =	Female =	

**What drives the decision to wean?**	(Age/ Weight etc)				
**Is dung removed from pens regularly and how often?**					
**How often are calf pens cleaned out entirely?**					
**What drives that decision?**					
**Are calves moved when cleaning out? If so, where? (eg. empty pens)**					
**When pens are cleaned out are they:**	Cleaned out only	Cleaned out and washed	Cleaned out and disinfected	Cleaned out, washed and disinfected	Other ____________
**Name of disinfectant if used: (Include label)**		***Amount/application:***			
**Name of desiccant powder if used: (Include label)**		***Amount/application:***			
**Comment:** *(e.g. does the disinfectant treat for the prevalent pathogens, where do they get their info from, why selected conc rate, how do they know it is the right product, do they ask vet?)*					

**Are pens easy to clean?** (use score below)	1	2	3	4
	n= ______	n= ______	n= ________	n = ___________
If a variety of responses are required, indicate how many pens fall into each category	1 = smooth surfaces, generally without tight gaps between frame and fittings, looks clean<br>2= surfaces are obviously cleaned 100%, but 10% of surface areas are pitted or corroded.<br>3=surfaces show signs of cleaning but more than 10% has engrained biofilm and some evidence of very difficult to clean areas.<br>4= visual evidence of lack of easy clean, thick biofilm on surfaces at calf height that has persisted across repeated 'cleanings'			

CALF HOUSE SKETCH ON PROVIDED GRIDLINED PAPER

Main Calf Building

**Outer building walls**	Timber	Concrete	Other ____________

**Roof materials**	Mineral Fibre	Tin	Other ______________		
**Pen walls**	Timber	Concrete	Plastic	Rubber	Other ____________
**Floor**	Mesh	Slats	Concrete	Other ________________	
**General cleanliness score** (use score below)	1	2	3	4	
**PHOTOS OF 3 PENS**	1 = easy clean surfaces, few difficult to reach areas<br>2 = mostly easy to clean, some evidence of residues<br>3= obvious residues and biofilm, broken surfaces at animal height<br>4 = broken surfaces at animal height, evidence of lack of cleaning				
**Proximity from calf house to other buildings**	__________________ m **(SKETCH ON DIAGRAM)**				
**Spirit Level data**	*Within 3 pens in the main calf house where there is easy access to the floor surface, measure the slope of the floor in representative locations*				
**Adequate pen drainage score** (use score below)	1	2	3	4	
	n =__________	n =___________	n =___________	n =___________	
If a variety of responses are required, indicate how many pens fall into each category	**1 = well draining, mostly devoid of small cracks in surface**<br>**2 = mostly well draining, some cracking in floors and at joint to wall**<br>**3= evidence of poor drainage ability, cracking in surfaces**<br>**4= significant poor drainage, broken surfaces, porous in parts**				
**Floor condition across building**	Rough	Smooth	Other ______________________		
**Main Bedding type**	Straw	Sawdust	Woodchip	Other ___________________	
**% of pen bedded**	100%	75%	50%	<50%	Slats
	n=________	n=________	n=________	n=________	n=________
**Nesting scores of calves in main calf building** **See Visuals**	1	2	3		
	n=____________	n=____________	n=____________		
**Kneel Test** *(Kneel with paper towel on bedding for 30 seconds – Are knees wet or dry?)* **See Visuals**	Dry	Wet			
**Bedding condition score** (use score below)	1	2	3	4	
	n =___________	n =___________	n =___________	n =___________	

If a variety of responses are required, indicate how many pens fall into each category	**1 = 95% of calves on a dry bed, clean calves**<br>**2 = 75% of calves on dry bed**<br>**3 = 33% (1/3rd) of pen surface damp**<br>**4 = as 3 above, but calves exposed to standing liquid**
**Comment:** (Using an alternative bedding due to price this year? Other observations)	
**Area of damp concrete in pen**	____________________% **(DIAGRAM)**
**Area of damp concrete outside pen**	____________________% **(DIAGRAM)**<br>*I.e. area under the same roof*

Ventilation

**Inside Temp °C**		**Inside R Humidity %**		**Inside Wind speed m/s**	

**What type of ventilation is used (Tick all that apply)**	Natural	Mechanical

**Condensation signs? PHOTOS**	Cobwebs			Dark Staining visual evidence of some staining of roof purlins			Stained Purlins Condensation present on underside of roof		
	Y	N	%	Y	N	%	Y	N	%

**Does the farmer think they use straw excessively?**	Y	N	**Dirty Calves?**	Y	N	**Straw Choppers used when bedding up?**	Y	N

**Smoke Test – time for smoke to disperse out of building (>5mins/ <5mins)**	**House 1**		**House 2**		**House 3**	

Comments - *where cobwebs, or staining on the purlins, or condensation on the underside of the roof are present, make an assessment of the percentage or fraction of the total roof area affected. This is easiest done by referring to the number of half bays (ie each side of the roof ridge) or parts thereof that are affected (ie 3 half bays in 4 bay building would be 3/8ths affected, or 37% :*

Main Calf Facility

**Is there a smell of damp?**	Y	N	**Are there traces of ammonia?**	Y	N
**Under what conditions does it get smelly in the calf house?**					
**Are post wean animals sharing the same air space? (3 months +)**	Y	N	**Are there adjoining buildings with livestock?**	Y	N
**Inlet/Outlet Areas**	**Types of Inlets and Outlets (DIAGRAM & PHOTOS)**				
**Is there dirty air spilling in from adjacent poorly ventilated spaces with older cows?**	Y	N	**Are fans used?**	Y	N
**Under what conditions are the fans turned off, if used?**					
**Where is the air intake of fans?**	Inside		Outside		
**Is the air distributed through a duct?**	Y	N	**If duct is used, what is the airspeed at animal height?**	____________m/s	
**Max number of animals in house at any one time**			**Calculate air space/ inlet area per calf**		
**Calf jackets?**	Y	**N**	**When used?**	What age of calf/type of calf gets a jacket?	
			**When taken off?**		
			**When Washed?**		
**Heat Lamps?**	Y	N	**Protocol?** *Ie when used and why*		
**Light level in calf pen with lights on** *At calf height in 3 pens across the building (hold light meter horizontal)*	________Lux<br>________Lux<br>________Lux		**Light level in calf pen with lights off**	____________Lux<br>____________Lux<br>____________Lux	
**% of Roof natural lighting**	%		**Number of lights in building**		

Veterinary health status in the last 3 years

Disease Status	Yes	Tested No	Don't Know	Vaccinate
IBR				Y / N
BVD				Y / N
Rotavirus				Y / N
Coronavirus				Y / N
Salmonella				Y / N
Leptospirosis				Y / N
E. Coli K99				Y / N
Mycoplasma Bovis				

**Vaccinate Calves <12 weeks for respiratory disease**	Yes	No
**Comments:**		

**Closed Herd status (Don't buy any animals including bulls)**	Y	N	**Visitors given access to calving pens or calf housing**	Y	N
**Foot dip present for farm yard PHOTOS**	Y	N	**How often is it replenished?**		
**Foot dip present for calf rearing area PHOTOS**	Y	N	**How often is it replenished?**		

Feeding

**Is there a cleaning area available for buckets etc in the calf house (not on the floor) PHOTOS**	Y	N	**Is there a drying area also available (not on the floor) PHOTOS**	Y	N
**Milk feeding method (Tick all that apply)**	Automatic Feeder	Single teat bucket	Multiple teat bucket	Bucket	Trough

**How often are the teat buckets cleaned?**	After every feed	Daily	Weekly	Other __________	
**Is equipment washed in:**	Cold water	Cold water and chemical	Hot water	Hot water and chemical	Other ________
**On automatic Feeders how often are the teats cleaned?**	Daily	Weekly	Monthly	When needed	Other_______
**Is equipment washed in:**	Cold water	Cold water and chemical	Hot water	Hot water and chemical	Other ________
**On automatic Feeders how often are the teats changed?**	Daily	Weekly	Monthly	Between seasons	Other_______
**How often is the Automatic feeder calibrated?**	Weekly	Monthly	Between seasons	Other_____________	
**Is the calf house used all year round?**	Y	N	**If yes, what is it used for?**		
**At what age are calves grouped, if individual and why?**					
**If you have a sick calf do you isolate it from others to prevent cross contamination?**	Y	N	**How do you manage sick calves?**	*(Ie. Is calf isolated immediately? How are they isolated? Are hygiene protocols put in place between sick and healthy calves? Heater or jacket?)*	
**At what point do you isolate?**					
**If milk is fed fresh, how is stored prior to feeding**	Fridge	Covered container cooled	Uncovered container cooled	Covered container uncooled	Uncovered container uncooled
**Is your milk pasteurised?**	Y	N	**Milk feeding plan in place?**	Y **(Fill in plan in appendix)**	N	Intuitively
**At what age do you first feed milk replacer**	________ days	**What brand of milk replacer do you use?**	Brand___________________________ **(include sample label)**		

<table>
<tr><td>What amount of milk powder to water do you use?</td><td colspan="5">__________________g X __________________L</td></tr>
<tr><td>Weight check (Powder)</td><td colspan="2">Assessor weight check 1</td><td colspan="3">Assessor weight check 2</td></tr>
<tr><td>Mixing temperature of milk replacer</td><td>Known/ Guessed</td><td>Not heated</td><td><40°C</td><td>40-65°C</td><td>>65°C</td></tr>
<tr><td>Actual temperature</td><td colspan="5">__________________°C (Measure with thermometer)</td></tr>
<tr><td>How long does it take from the first calf fed to the last calf fed?</td><td colspan="5">Currently __________ hours
Time for 1 feed, not including a second feed in the same day.</td></tr>
<tr><td>Name and manufacturer of concentrate</td><td>______________________________
(Include sample label)</td><td>Protein content</td><td>%</td><td>Oil Content</td><td>%</td></tr>
<tr><td>Water Origin</td><td colspan="2">Own source/ well</td><td colspan="3">Mains</td></tr>
<tr><td>Conductivity (measures low concs of nutrients, minerals and dissolved solids) Troughs & Buckets

Recalibrate sensor before every visit</td><td>Pen 1
(Individ./ Group)</td><td>Pen 2
(Individ./ Group)</td><td>Pen 3
(Individ./ Group)</td><td colspan="2">Pen 4
(Individ./ Group)</td></tr>
<tr><td colspan="6">Conductivity samples. Rinse sample head in clean water and remove excess water with tissue between samples.
Measure sample from the centre of the water volume in each trough/drinker/bucket.</td></tr>
<tr><td>Water Flow Rate [1]

Troughs only

Volume/minute or seconds</td><td>Pen 1
(Individ./ Group)</td><td>Pen 2
(Individ./ Group)</td><td>Pen 3
(Individ./ Group)</td><td colspan="2">Pen 4
(Individ./ Group)</td></tr>
<tr><td colspan="6">[1]Note or mark the high water level in trough.
Dip plastic measuring jug into water and remove as much water in one go as is practicable, start stop watch immediately; - stop the watch when flow rate into trough starts to decline when trough almost full.
Flow rate is volume of water in jug/stop watch time in seconds.</td></tr>
</table>

Where drinker is too small for jug, put large plastic bag around drinker, depress/activate the float valve to start normal water flow. When water overflows the drinker and starts to flow into the bag, immediately start the stopwatch and collect water for 15 seconds. Volume collected x 4 is flow rate per minute.				
**Water Sample** [2]<br>*Refer to protocol in appendix*	Pen 1<br>(Individ./ Group)<br>Y / N	Pen 2<br>(Individ./ Group)<br>Y / N	Pen 3<br>(Individ./ Group)<br>Y / N	Pen 4<br>(Individ./ Group)<br>Y / N

**Water facilities**<br>*If pens 1-4 have different access, hygiene etc, note scores in row below*	Access				Hygiene				Cleanability			
	1 = Good ht, simultaneous drinking<br>2 = Good ht. single drinking<br>3 = Restricted access height<br>4 = Restricted access; any				1 = clean visual and smell<br>2 = some sedimentation<br>3 = sediment, surface debris<br>4 = Gross contam. & faeces				1 = front of pen, drain plug<br>2 = front of pen, no drain<br>3 = not front of pen, clean<br>4 = not front of pen, dirty			
	1	2	3	4	1	2	3	4	1	2	3	4

**Comments**

Main Calf Rearer

**Age**		**Sex**	M / F
**Number of years working in the agriculture industry?**	___________________ Years		
**Do you currently have an off farm job?**	Yes	No	
**Hours currently spent feeding calves per day?**	___________________hours	(Milk, Concentrate, Forage)	

**Hours currently spent cleaning the calf house, equipment etc. per day?**	___________________hours
**Hours currently spent per day doing other calf house related tasks?**	___________________hours
**Days per week spent working on the farm?**	___________________days
**What do you like about your job?**	
**Any dislikes about your job?**	
**Have you made any changes to your calf rearing system in the last 3 years?**	
**Is there anything you would like to change? (If not done, why not?)**	
**Any Training received?**	

<u>Second calf Rearer</u>

**Age**		**Sex**	M / F
**Number of years working in the agriculture industry?**	___________________ Years		
**Do you currently have an off farm job?**	Yes	No	
**Hours currently spent feeding calves per day?**	___________________hours	(Milk, Concentrate, Forage)	

**Hours spent cleaning the calf house, equipment etc. per day?**	____________________hours
**Hours spent per day doing other calf house related tasks?**	____________________hours
**Days per week spent working on the farm?**	____________________days
**What do you like about your job?**	
**Any dislikes about your job?**	
**Any Training received?**	

**Main Sources of Information (Rank top 3 sources)**	**Main Calf Rearer**	**Second Calf Rearer**
**Conferences/ Farm walks**		
**Booklets and Manuals**		
**Farming Press**		
**Internet**		
**Vet**		
**Family Member**		
**Other Farmers**		
**DAERA Advisors**		
**Company Representatives**		
**Television/ Radio**		
**Agricultural Shows**		
**Discussion Groups**		
**Short Courses**		
**Main calf rearer**		
**Other ________________________**		

Milk Feeding Plan

Days of age	Feed frequency	L/calf/ day	g/calf /Day	Concentrate (Kg/day)	Forage Type	Forage Introduced	Water access
**0**	Once a day/ Twice a day/ Ad Lib					Rack/ Trough/ None	Bucket/ ad lib/ None
**7**	Once a day/ Twice a day/ Ad Lib					Rack/ Trough/ None	Bucket/ ad lib/ None
**14**	Once a day/ Twice a day/ Ad Lib					Rack/ Trough/ None	Bucket/ ad lib/ None
**21**	Once a day/ Twice a day/ Ad Lib					Rack/ Trough/ None	Bucket/ ad lib/ None
**28**	Once a day/ Twice a day/ Ad Lib					Rack/ Trough/ None	Bucket/ ad lib/ None
**35**	Once a day/ Twice a day/ Ad Lib					Rack/ Trough/ None	Bucket/ ad lib/ None
**42**	Once a day/ Twice a day/ Ad Lib					Rack/ Trough/ None	Bucket/ ad lib/ None
**49**	Once a day/ Twice a day/ Ad Lib					Rack/ Trough/ None	Bucket/ ad lib/ None
**56**	Once a day/ Twice a day/ Ad Lib					Rack/ Trough/ None	Bucket/ ad lib/ None
**63**	Once a day/ Twice a day/ Ad Lib					Rack/ Trough/ None	Bucket/ ad lib/ None
**70**	Once a day/ Twice a day/ Ad Lib					Rack/ Trough/ None	Bucket/ ad lib/ None
**77**	Once a day/ Twice a day/ Ad Lib					Rack/ Trough/ None	Bucket/ ad lib/ None
**84**	Once a day/ Twice a day/ Ad Lib					Rack/ Trough/ None	Bucket/ ad lib/ None

Calving Profile

August 2018 – August 2019

Month	Aug 18	Sep	Oct	Nov	Dec	Jan	Feb	Mar	Apr	May	Jun	Jul 19
No. of Cows calved												
No. of viable calves at 48hrs												
Calf mortality (2 days to weaning)												
No. of calves treated for scour												
No. of calves treated for pneumonia												
Comments:												
